# Functional Consequences of Differential *O*-glycosylation of MUC1, MUC4, and MUC16 (Downstream Effects on Signaling)

**DOI:** 10.3390/biom6030034

**Published:** 2016-07-30

**Authors:** Ryan L. Hanson, Michael A. Hollingsworth

**Affiliations:** Eppley Institute for Research in Cancer and Allied Diseases, University of Nebraska Medical Center, Omaha, NE 68198, USA; ryan.hanson@unmc.edu

**Keywords:** mucin, cancer, *O*-glycosylation, MUC1, MUC4, MUC16, signaling

## Abstract

Glycosylation is one of the most abundant post-translational modifications that occur within the cell. Under normal physiological conditions, *O*-linked glycosylation of extracellular proteins is critical for both structure and function. During the progression of cancer, however, the expression of aberrant and truncated glycans is commonly observed. Mucins are high molecular weight glycoproteins that contain numerous sites of *O*-glycosylation within their extracellular domains. Transmembrane mucins also play a functional role in monitoring the surrounding microenvironment and transducing these signals into the cell. In cancer, these mucins often take on an oncogenic role and promote a number of pro-tumorigenic effects, including pro-survival, migratory, and invasive behaviors. Within this review, we highlight both the processes involved in the expression of aberrant glycan structures on mucins, as well as the potential downstream impacts on cellular signaling.

## 1. Mucins: Structure and Function

Mucins, a large family of glycoproteins, are expressed by epithelia of the respiratory, gastrointestinal, and reproductive tracts [1]. Consisting of high molecular weight glycoproteins, mucins are broadly classified as secretory or membrane bound. Secretory mucins coat the epithelial surface and provide a protective molecular barrier that is appropriate for the specialized epithelia by which it is produced. Membrane bound mucins are generally localized to the apical surface of epithelial cells by a transmembrane domain and cytoplasmic tail, which is known to engage in signal transduction events [1,2]. Together these mucins make up a significant proportion of the proteins found in the mucosal layers of most tissues in the aero-digestive tract [2,3,4,5]. The secretory family of mucins includes mucin 2, oligomeric mucus/gel-forming (MUC2), mucin 5AC, oligomeric mucus/gel-forming (MUC5AC), mucin 5B, oligomeric mucus/gel-forming (MUC5B), mucin 6, oligomeric mucus/gel-forming (MUC6), and mucin 7, secreted (MUC7), whereas, mucin 1, cell surface associated (MUC1), mucin 3A, cell surface associated (MUC3A), mucin 3B, cell surface associated (MUC3B), mucin 4, cell surface associated (MUC4), mucin 12, cell surface associated (MUC12), mucin 13, cell surface associated (MUC13), mucin 15, cell surface associated (MUC15), MUC16, mucin 17, cell surface associated (MUC17), and mucin 20, cell surface associated (MUC20) are membrane bound [2]. Under normal physiological conditions, mucins play an essential role in lubrication, chemical sensing, and molecular configuration of the local cellular microenvironment [1]. In addition to forming a protective barrier, mucins are postulated to act as sensors of surrounding environmental conditions [6,7,8]. Transmembrane mucins are known to associate with receptors and other kinases that phosphorylate specific residues, enabling their association with signaling proteins and transcription factors, which in turn apprises the cell of molecular and morphogenetic conditions at the cell surface and accordingly reprograms RNA and protein expression [8].

One defining structural characteristic of mucin proteins is the presence of a tandem repeat domain or mucin domain [2]. The amino acid sequence and number of these repeats varies, but they are universally rich in serine, threonine, and proline residues that form multiple potential sites for *O*-linked glycosylation [9,10]. *O*-glycosylation is critical for mucin function, as *O*-linked oligosaccharides confer specific molecular features that modulate ligand-receptor interactions and biochemical properties critical for organization and function of the extracellular environment [11,12]. As the number and sequence of the tandem repeats is highly variable, mucins present a wide array of potential glycosylation patterns.

**Figure 1:**
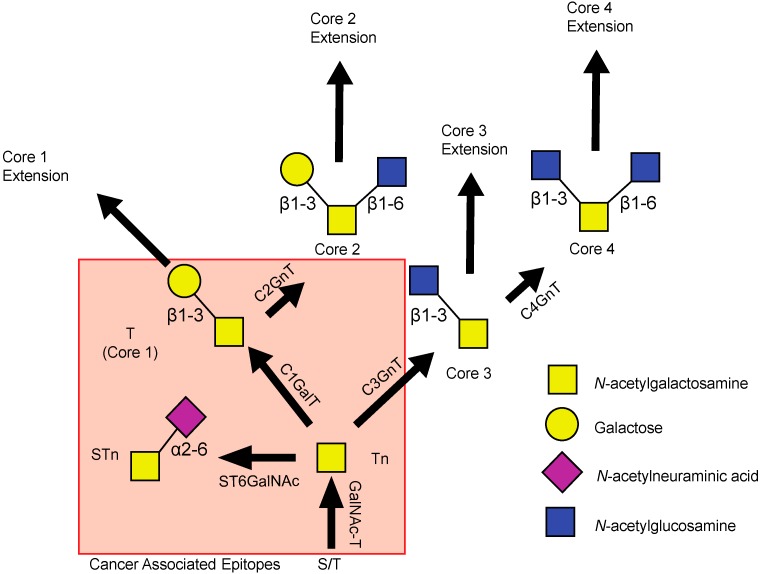
*O*-type glycosylation of mucins. Schematic representation of mucin *O*-type glycosylation. Initiation occurs through addition of *N*-acetylgalactosamine (GalNAc) to serine or threonine residues present in the mucin backbone. These structures are then extended into Core 1, Core 2, Core 3, and Core 4 structures through the addition of the indicated sugar. The enzyme involved in each reaction is indicated with the arrow and linkage lines indicate the attachment for each sugar. The cancer associated epitopes T, Tn, and sialyl-Tn (STn) are highlighted within the box. Gal: galactose; GalNAc-T: GalNAc-transferase; C1GalT: Core 1 Gal-transferase; C2GnT: Core 2 *N*-acetylglucosamine transferase; C3GnT: Core 3 *N*-acetylglucosamine transferase; C4GnT: Core 4 *N*-acetylglucosamine transferase.

The process by which mucin type *O*-linked glycosylation occurs is well characterized, though we know relatively little about its regulation [13,14,15,16] (Figure 1). The initiating step involves the addition of *N*-acetylgalactosamine (GalNAc) to serine or threonine residues present in the mucin backbone to form the Tn-epitope, a step that is catalyzed by a large family of polypeptide GalNAc-transferases (GalNAc-Ts) [13,17]. These structures can then be further extended to form Core 1, 2, 3, or 4 structures based on the identity of the carbohydrate and linkage [18]. Core 1 structures are formed by addition of galactose (Gal) in a β1-3 linkage to GalNAc, which is catalyzed by a single enzyme, Core 1 Gal-transferase (C1GalT1) [19]. Core 1 structures can be extended or Core 2 structures can be generated by addition of *N*-acetylglucosamine (GlcNAc) in a β1-6 linkage to the existing GalNAc of the Core 1 structure by Core 2 GlcNAc transferases (C2GnTs) [20,21,22]. As an alternative to Core 1, Core 3 structures can be generated through addition of GlcNAc in a β1-3 linkage to the Tn epitope [23]. Like Core 1 structures, Core 3 structures may be extended or act as the scaffold for Core 4 structure generation through addition of another GlcNAc in a β1-6 linkage [22]. While other core structures do exist, Core 1, 2, 3, and 4 structures comprise the primary glycan structures observed in humans.

## 2. Deregulation of Mucin Expression and *O*-Type Glycosylation in Cancer

Deregulated expression of mucins is observed in many malignancies; particularly within certain tumor types (Table 1). Elevated expression of MUC1 is common in pancreatic, breast, colon, lung, and prostate cancer [24,25,26,27]. Similarly, MUC4 expression is increased in colon adenocarcinoma samples and is a proposed marker of aggressive pancreatic cancer [24,28]. Elevation of MUC16 (CA125) is well studied in ovarian cancer, and recently expression of MUC16 has been implicated as a significant factor in pancreatic cancer [29,30,31,32].

Many tumors exhibit aberrant *O*-glycans. Alterations in the glycobiology of tumors occur principally through two mechanisms: neo-synthesis and incomplete synthesis [44]. The expression of truncated Core 1 based structures, such as T, Tn, or sialyl-Tn (STn), are observed in a majority of human carcinomas. These structures are typically absent in healthy tissues [24,33,45]. In many instances, expression of these truncated structures is driven by alterations to the expression of enzymes involved in the glycosylation process. For example, the extension of Core 1 structures relies on a single enzyme, C1GalT1. This enzyme requires a specific chaperone, Core 1 β3GalT specific molecular chaperone (Cosmc), for proper folding and functional activity [46,47,48,49]. Cells lacking expression of Cosmc have been shown to exhibit increased levels of Tn and STn epitopes [46,50,51]. Furthermore, a significant percentage of cancers exhibit hypermethylation of the *Cosmc* gene, resulting in decreased expression and increased formation of Tn and STn epitopes [52,53]. Another potential factor is deregulation of enzymes that extend or terminate extension of *O*-glycans (e.g., sialyl transferases), which has also been observed in a variety of cancers [54,55,56,57,58,59]. This may explain observed decreases in expression of Core 3 and 4 structures in gastric and colorectal cancers [60,61].

Recent studies have also found that the localization of GalNAc-Ts is a critical factor in the generation of *O*-glycan structures [62,63]. Relocation of GalNAc-Ts from the Golgi to the endoplasmic reticulum (ER) results in changes to the compartmentalization of the initiation machinery and the normal *O*-glycosylation process. Interestingly, this shift in localization appears to be dependent on proto-oncogene tyrosine-protein kinase Src (Src) activity [62]. Redistribution of GalNAc-Ts has been shown to result in increased density of GalNAc modification within a six tandem repeat model of MUC1, indicating a role for this process in the glycosylation of mucins [63]. Proteomic analysis has also demonstrated that the density of *O*-glycosylation is increased on MUC1 secreted from breast cancer cells [64]. Furthermore, high density GalNAc modification is associated with increased aggressiveness in breast cancer [65]. The density of GalNAc modifications can regulate the in vitro activity of core extension enzymes suggesting that the redistribution of GalNAc-Ts to the ER may promote increased formation of truncated glycans [66]. The role of Src activity in metastatic behavior further highlights a potential link between the expression of truncated glycans, GalNAc-T localization, and an aggressive tumor phenotype [52,62,63,67].

Another contributing factor may be alterations in mucin core protein levels that were discussed in the preceding paragraph. Overexpression or altered expression of protein acceptor substrates (such as mucin tandem repeat domains) may saturate the catalytic machinery of glycosylation in some cells and lead to incomplete extension or premature truncation of some glycans. Alterations in levels of acceptor substrates for *O*-glycans (mucin core proteins) in tumor cells may also explain in part the observed increased expression of some glycoepitopes, such as sialyl-Lewis^X/A^, which are commonly observed in adenocarcinomas [59]. While there is little evidence for this occurring in physiological conditions, systems used for production of recombinant proteins do show evidence for the overwhelming of machinery involved in the processing of secreted and membrane bound proteins [68,69].

**Figure 2:**
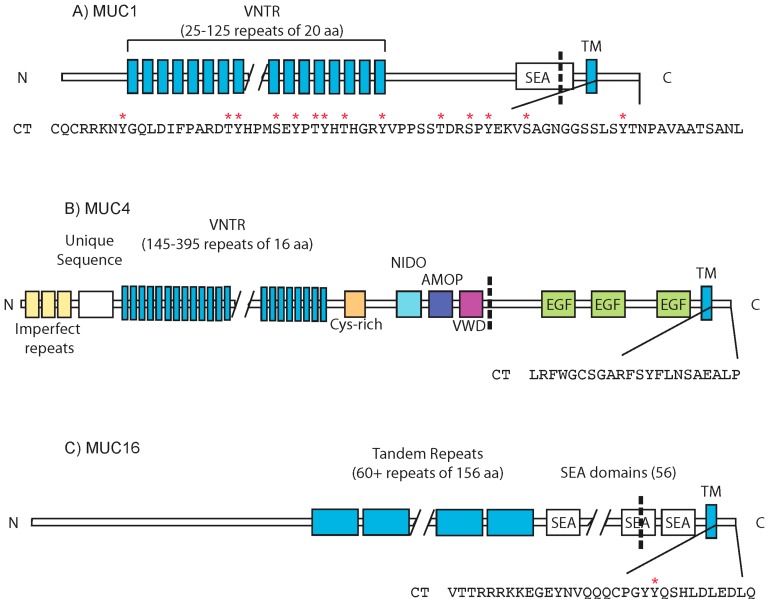
Structure of MUC1, MUC4, and MUC16. General domain structures for MUC1 (**A**); MUC4 (**B**); and MUC16 (**C**). Cleavage sites are represented by dashed lines and the sequence of the cytoplasmic tail is presented for each mucin. Confirmed phosphorylated residues are indicated by red asterisks (*). Proteins are not drawn to scale. VNTR: variable number tandem repeat domain; SEA: sperm protein, enterokinase, agrin domain; TM: transmembrane domain; CT: cytoplasmic tail; NIDO: nidogen-like domain; AMOP: adhesion-associated domain in MUC4 and other proteins; VWD: Von Willebrand factor type D domain; EGF: epidermal growth factor-like domain.

Phenotypically, altered expression of mucin-type glycoproteins bearing aberrant *O*-glycans is associated with increased aggressiveness and metastatic behavior in a variety of cancers [33,52,59]. These effects result in part from changes to binding properties of secreted and cell surface proteins that modulate interactions between tumor cells and binding partners in the extracellular environment (e.g., selectins and integrins) [59] and from effects on other ligand-receptor interactions that alter signal transduction in affected cells. Re-expression of enzymes involved in the extension of the carbohydrate chain, such as Cosmc or Core 3 synthase, results in a decrease in these aggressive properties in pancreatic cancer cells by influencing these interactions [70]. Within this review, we discuss potential molecular mechanisms whereby alterations in mucin type *O*-glycosylation mediate functional effects, particularly in regards to modulation of downstream signaling through the cell surface mucins MUC1, MUC4, and MUC16 (Figure 2).

## 3. Signaling Through the Cytoplasmic Tail

### 3.1. MUC1

MUC1 consists of two distinct subunits: a large N-terminal extracellular domain that contains a variable number tandem repeat (VNTR) domain and a shorter C-terminal fragment consisting of a short extracellular domain, a transmembrane domain, and a cytoplasmic tail (MUC1.CT) (Figure 2A). Following translation, MUC1 undergoes an auto-proteolytic event within the sperm protein, enterokinase, agrin (SEA) domain and exists at the cell surface as a heterodimer of these two subunits [71,72,73]. Signaling through the MUC1 cytoplasmic tail, although the most characterized among the transmembrane mucin family members, remains poorly understood.

Given that MUC1 is distributed at the apical surface of normal epithelia, it has been our longstanding hypothesis that a principal function of MUC1 morphogenetic signaling is to assist in reprogramming gene expression in response to alterations in cell morphology (such as loss of cell polarity). MUC1 also functions in the context of other stimuli, such as the presence of cytokines or growth factors that may be produced during tissue damage, inflammation, or tissue remodeling [1]. Integration of signaling is accomplished by differential phosphorylation of specific residues within the 72 amino acid cytoplasmic tail [8,74,75]. Phosphorylation is mediated through the interaction of MUC1 with specific receptor tyrosine kinases (RTKs) at the cell surface, including hepatocyte growth factor receptor (Met), epidermal growth factor receptor (EGFR), or platelet-derived growth factor receptor β (PDGFRβ) [74,76,77], MUC1 can be phosphorylated on serine residues by glycogen synthase kinase 3β (GSK3β) and mass spectrometry assays have shown phosphorylation on other serine and threonine residues [78,79,80,81]. The cytoplasmic tail of MUC1 contains 22 potential sites of phosphorylation (seven tyrosines, nine serines, and six threonines) allowing for a wide array of potential phosphorylation patterns. The specific patterning of the phosphorylated sites is hypothesized to specify association of MUC1 with different downstream effectors, including growth factor receptor-bound protein 2 (GRB2)/son of sevenless (SOS), to initiate downstream signaling cascades [82,83,84,85,86]. Of these 22 sites, the majority have been demonstrated to be phosphorylated under various conditions (Table 2). The precise function of many phosphorylation sites, however, remains unknown. MUC1 can also translocate to the nucleus where it functions as a transcriptional co-regulator in association with transcription factors such as β-catenin and p53 [74,75,87,88,89,90].

The effect of glycosylation on the interactome of MUC1 and the resulting impact on phosphorylation, signaling, and downstream effectors has only recently begun to be explored in depth. As many of MUC1’s interaction partners reside in the extracellular compartment, any alterations to the extensive carbohydrate chains may result in significant changes to the overall structural conformation of the extracellular domain. Loss of branching glycans may preferentially promote interactions by exposing ligand-binding sites or may inhibit binding through the loss of glycan specific interactions, such as lectin-like binding sites. In a fully glycosylated state, MUC1 may also sequester factors and prevent them from reaching activating receptors. Loss of glycosylation could potentially result in loss of this sequestration, increasing local concentrations of these factors to alter downstream signaling.

Loss of Core 1 derived glycans through knockout of C1GalT1 in a mouse model of breast cancer was shown to decrease the incidence of tumor development [98]. While, presumably, loss of C1GalT1 should favor formation of truncated glycans and tumor progression, loss of Core 1 glycans may favor formation of Core 3 or 4 structures that correlate with less aggressive tumors [60,61,70]. This model also disrupted MUC1 expression and impacted downstream effectors, including extracellular signal-regulated kinase (ERK), RAC-alpha serine/threonine-protein kinase (AKT), and phosphoinositide 3-kinase (PI3K) activation [98]. This may also account for the observed decrease in tumor incidence. Conversely, overexpression of C1GalT1 in breast cancer cells increased association between MUC1 and β-catenin by promoting the shedding of the extracellular domain [99], which was correlated with increased migratory and invasive behavior. Loss of the extracellular domain of MUC1 may promote a conformational change within MUC1 to promote this interaction. Overexpression of C1GalT1 may also potentiate increased formation of T structures, as well as potential extension to form sialyl-Lewis moieties associated with metastasis. Interestingly, overexpression of MUC1 in human breast cancer lines as well as murine lines results in decreased expression of the extension enzymes core 2 β1,6-*N*-acetylglucosaminyl transferase 1 (C2GnT1) and ST3 β-galactosidase α-2,3-sialyl transferase 1(ST3Gal1) suggesting that MUC1 can potentiate expression of truncated glycans in a feed forward manner [100].

Additional studies in breast cancer demonstrate that hypoglycosylation of MUC1 to form Tn and STn antigens results in increased association with the SH3 domain-containing kinase-binding protein 1, CIN85. This association results in increased migratory and invasive properties [101]. While the precise downstream signaling mechanisms are unknown, CIN85 contributes to endocytic trafficking of activated receptor tyrosine kinases, including EGFR [102,103]. Interestingly, the association of MUC1 and EGFR has been shown to result in the nuclear translocation of the complex and increased expression of cyclin D1 [86]. There is evidence that MUC1 may directly regulate cyclin D1 mRNA levels by interacting with β-catenin and p120 catenin, thus integrating Wnt signaling with epidermal growth factor (EGF) signaling in some cell types [88]. MUC1 is also known to potentiate downstream signaling through both the ERK and AKT signaling cascades [104,105]. The activation of upstream signaling cascades by MUC1 can have significant impacts on the activation and regulation of downstream transcription factors resulting in reprogramming of gene expression profiles to favor tumor progression [85,106,107]. Furthermore, galectin-3, whose binding affinity is altered depending on the structural glycans on MUC1, regulates the association between both MUC1 and EGFR [108,109,110,111]. These results suggest that altered MUC1 glycosylation may readily promote the association of MUC1 and EGFR and also integrate morphogenetic signals from the Wnt pathway. This in turn promotes endocytosis of the complex by CIN85, and alters the compartment of signaling to drive oncogenic effects in tumor cells.

In addition to regulating the association of MUC1 and EGFR, galectin-3 may also regulate the density of the mucin barrier surrounding cells. In A375 melanoma cells, overexpression of MUC1 inhibits adhesion to endothelial cells, however, addition of galectin-3 results in increased adhesion [112]. Galectin-3 modulates the exposure of adhesion molecules, such as CD44, which are normally masked by the dense barrier created by MUC1 [112,113]. These effects may explain in part the evidence that MUC1 plays both an adhesive and anti-adhesive role within cancer. With high-density mucin expression, epitopes involved in adhesion are masked, whereas presence of galectin-3 disrupts the dense barrier and exposes these molecules resulting in enhanced adhesion. These effects would allow tumor cells to disseminate through the body before adhering to distal sites to form metastatic colonies. Expression of galectin-3 also promotes cell aggregation to allow tumor cells to avoid anoikis [113]. While these studies have focused solely on the role of galectin-3, it is possible that truncated glycan structures may also disrupt the dense barrier surrounding tumor cells independent of galectin-3. As such, modulation of glycan length may play a critical role in balancing adhesive and anti-adhesive features in the absence of galectin-3.

**Figure 3:**
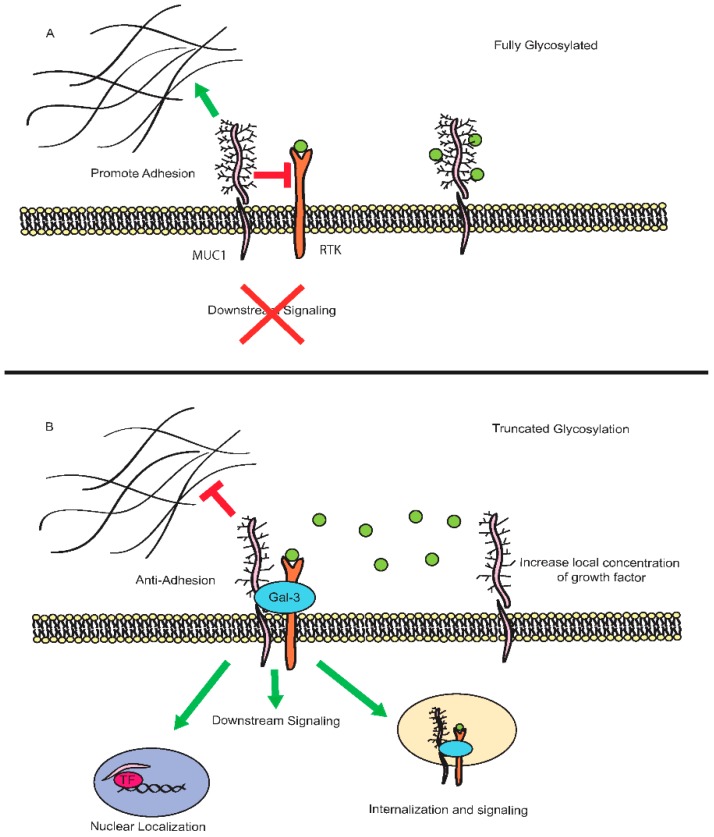
Impact of *O*-glycosylation on MUC1 signaling. In fully glycosylated state (**A**), the interaction of MUC1 with signaling partners, such as epidermal growth factor receptor (EGFR), may be enabled or inhibited either through steric effects or by masking of interaction domains. Glycosylation may also sequester growth signals and decrease availability for receptor mediated signaling. Branched glycans may also promote adhesive effects of MUC1 and inhibit migration. Loss of glycosylation (**B**) can promote association between MUC1 and signaling partners, either through direct interactions or those mediated by adaptor partners, like galectin-3 (Gal-3). These interactions can promote downstream signaling from the surface, or internalization of the complexes to compartmentalize signaling. Loss of glycosylation also promotes anti-adhesive behavior through interactions in the microenvironment and may result in loss of capacity to sequester growth signals. RTK: receptor tyrosine kinase; TF: transcription factor.

Studies have also demonstrated that the internalization of MUC1 by clathrin-mediated endocytosis is regulated by its glycosylation state. In glycosylation-defective Chinese hamster ovary (CHO) cells, reduced expression of MUC1 is observed at the plasma membrane [114]. Interestingly, this is the result of increased endocytosis, but does not result in increased degradation of internalized MUC1. This suggests that MUC1 may produce a prolonged signal within these intracellular compartments. Mutational analysis of specific tyrosine residues demonstrates that this internalization is dependent on the YHPM and YTNP sites within the MUC1 cytoplasmic tail [91]. These residues have been shown to be phosphorylated by a range of kinases, including Met, EGFR, and Src [2,74,115]. As such, decreased glycosylation of MUC1 may allow for increased interactions with these kinases, promoting internalization and compartmentalized signaling through MUC1. Association with other factors, such as p53 and β-catenin, may help to further localize MUC1 to the appropriate signaling compartment (Figure 3).

### 3.2. MUC4

MUC4 shares several structural similarities with MUC1; however, it also contains unique domains that confer distinct functions (Figure 2B). Similar to MUC1, MUC4 undergoes a proteolytic cleavage and exists as two fragments, MUC4α and MUC4β. However, the cleavage of MUC4 is apparently not mediated by an SEA domain, as MUC4 is the only transmembrane mucin lacking an SEA domain as identified by homology [116]. MUC4α contains several structural domains including a nidogen-like domain (NIDO), the VNTR, an adhesion-associated domain in MUC4 and other proteins (AMOP), as well as a cysteine-rich domain and a von Willebrand factor type D sequence (VWD) [2,116,117]. MUC4β has three EGF-like domains and a short cytoplasmic tail of just 22 amino acids [116,118], which contains potential sites of phosphorylation, although there have been no reported functional characterizations of phosphorylated residues within the MUC4 cytoplasmic tail to date.

**Figure 4:**
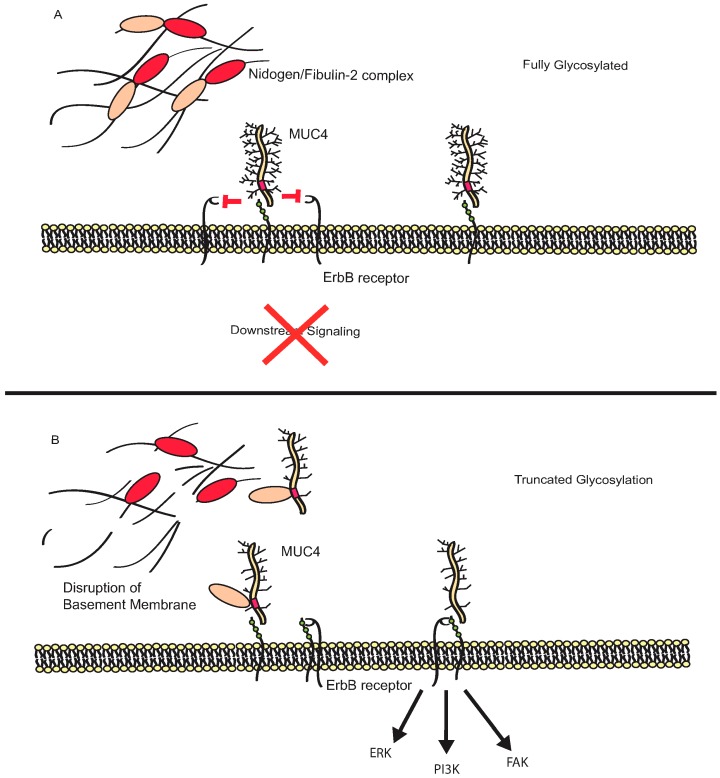
Impact of *O*-glycosylation on MUC4 signaling. In fully glycosylated state (**A**), MUC4 may not interact with Erb-B2 receptor tyrosine kinase (ErbB) family members, due to masking of epidermal growth factor (EGF)-like domains. Masking of nidogen-like (NIDO) domain also results in maintenance of basement membrane integrity. With loss of glycosylation (**B**), EGF-like domains may be exposed resulting in stabilization of ErbB signaling complexes and promotion of extracellular signal-regulated kinase (ERK), phosphoinositide 3-kinase (PI3K), and focal adhesion kinase (FAK) signaling. Exposure of the NIDO domain can also result in loss of basement membrane integrity through disruption of fibulin-2/nidogen complexes and promote invasive behavior.

MUC4 has primarily been shown to initiate signaling cascades through interactions with members of the ErbB family of receptors [119,120,121,122,123,124]. In particular, MUC4 potentiates downstream signaling through association with receptor tyrosine-protein kinase erbB-2 (ErbB2/HER2) and receptor tyrosine-protein kinase erbB-3 (ErbB3/HER3) [121,123]. The interaction of MUC4 with HER2 results in stabilization of the HER2/HER3 complexes and tyrosine phosphorylation of HER2. These interactions can potentiate signaling-induced programs of differentiation, cellular proliferation or inhibition of apoptosis, depending on the signaling context [123,125,126]. Furthermore, association of MUC4 and HER2 results in stabilization of the complex and can protect tumor cells from trastuzumab, a targeted therapy against HER2 [123,127]. Interestingly, only the EGF-like domains are required for the interaction of MUC4 with HER2, as the signaling cascades can be induced by forms lacking the mucin tandem repeat domain or the cytoplasmic tail [128]. Although interactions with EGFR members do not rely on the *O*-glycosylated extracellular domain of MUC4, it is possible that the decreased *O*-glycosylation associated with tumors may expose these EGF-like domains, allowing for potentiation of signaling through either HER2 or HER3 (Figure 4).

MUC4 also exhibits signaling capacity independent of ErbB2 suggesting the possibility of other signaling partners for this mucin. In HER2-low cells, MUC4 interacts primarily with HER3 and drives downstream activation of pathways involved in aggressive phenotypes, including activation of PI3K-, ERK-, and focal adhesion kinase (FAK)-associated pathways [121]. Expression of MUC4 also appears to regulate the localization of β-catenin by regulating the levels of E-cadherin within pancreatic cancer cells through activation of Src and FAK [129]. Recently, the AMOP domain of MUC4 has been demonstrated to play a role in the metastatic spread of pancreatic cancer cells [130]. These studies utilized the MUC4 splice variant MUC4/Y, which lacks the tandem repeat domain. As a result, MUC4/Y is likely less densely glycosylated and the functional domains in the core protein may be more readily accessible to interaction partners. The AMOP domain was also shown to be critical for the expression of vascular endothelial growth factor (VEGF)-A and matrix metallopeptidase (MMP)-9, which are both downregulated in response to MUC4 knockdown [129,130].

Beyond interactions with HER2 and HER3, MUC4 has been shown to associate with a number of extracellular matrix (ECM) proteins involved in the invasion and metastasis of tumor cells. Knockdown of MUC4 significantly increases the affinity of tumor cells to bind to laminin, collagen IV, and collagen I, among other ECM proteins [131]. Additionally, the NIDO domain of MUC4 has also been shown to play a critical role in the migration and invasion of tumor cells. This is postulated to result in part from inhibition of the normal interaction between nidogen and fibulin-2 proteins that control basement membrane integrity [132]. Given the potential role of the extended full-length form of MUC4 in the organization of the extracellular environment [1], it is likely that loss of normal glycosylation plays a substantial role in the alteration of these binding properties. Likewise, loss of glycosylation may expose the NIDO domain, allowing for increased association with fibulin-2 proteins and disruption of the normal extracellular organization.

### 3.3. MUC16

MUC16 is the largest mucin, with a core protein of roughly 22,000 amino acids and a molecular weight of 2.5 MDa. Glycosylation of the protein backbone further increases the mass to a predicted size of approximately 20 MDa [2,41,133,134]. Like other mucins, MUC16 contains several mucin-type tandem repeats, which are significantly longer than those of MUC1 and MUC4, and contain 156 amino acids [135]. MUC16 is predicted to contain 56 SEA domains, many of which are interspersed among the tandem repeats (Figure 2C). While most of these domains are distinct from other mucin SEA domains, the penultimate SEA domain has significant sequence conservation with the single SEA domain of other mucins, and is proposed to serve as a site of cleavage [116]. A second putative site of cleavage has also been proposed in the final SEA domain [135]. MUC16 contains a short cytoplasmic tail of 32 amino acids with several potential phosphorylation sites [2].

Interestingly, expression of as few as 114 amino acids from the C-terminal portion of MUC16 is sufficient to increase soft agar growth and the invasive properties of cancer cells [136], supporting a significant role for the signaling capacity of the C-terminal portion of MUC16. Following shedding of the larger extracellular domain of MUC16, this 114 amino acid fragment would remain in the cell membrane, where it may engage in signaling at the surface or undergo endocytosis to affect signaling in other cellular compartments. Like MUC1, the C-terminal portion of MUC16 has been shown to translocate to the nucleus in certain contexts and is present within the chromatin bound fraction, suggesting MUC16 may also function as a transcriptional co-regulator [96]. Disruptions in glycosylation may readily expose MUC16 for proteolytic cleavage of the extracellular domain, and thus potentiate signaling of the cytoplasmic tail to drive progression of cancer [137].

The cytoplasmic tail of MUC16 has been shown to interact with Janus kinase 2 (JAK2), resulting in increased proliferation, and an increase in signal transducer and activator of transcription 3 (STAT3) activity [138]. Expression of the 114 amino acid C-terminal form of MUC16 enhances the nuclear localization of JAK2 in pancreatic cancer cells to promote metastatic and stem-like properties [139]. This interaction is potentially mediated by the poly-basic sequence (RRRKK) of the MUC16 cytoplasmic tail that has been shown to interact with the ezrin/radixin/moesin family of proteins (ERM). JAK2 contains an ERM domain that is proposed to mediate the interaction of JAK2 with transmembrane proteins. As the interaction of MUC16 and JAK2 does not require the extracellular portion of MUC16, altered conformations that result from differential glycosylation or shedding of the extracellular domain may affect morphogenetic signaling by modifying association of the cytoplasmic tail and JAK2.

MUC16 also interacts with both Src and tyrosine protein-kinase Yes of the Src family of kinases [97]. This interaction results in phosphorylation of tyrosine 22142 in the cytoplasmic tail of MUC16, and promotes shedding of the extracellular domain. This phosphorylation of the MUC16 cytoplasmic tail may also lead to deregulation of β-catenin and E-cadherin at junctional complexes, as MUC16 has been shown to interact with both of these proteins [140]. The precise role of MUC16 in metastasis remains complex and poorly understood [97,138,139,140,141,142]. The action of Src on MUC16 may represent a confluence of signaling mechanisms, promoting the shedding of the extracellular domain, propagating a morphogenetic change in the structure of MUC16, and driving downstream activation of pathways driven by the interaction of the cytoplasmic tail with JAK2 or other potential partners.

## 4. Additional Roles for Aberrant Glycosylation in Tumor Progression

Beyond the capacity of glycosylation to alter downstream signaling through the cytoplasmic tails of mucins, these alterations can significantly influence the interactions of tumor cells with the surrounding microenvironment. One example is interactions with the immune system. Altered glycosylation of mucins often provokes immune responses in humans, as evidenced by the fact that many patients exhibit autoantibodies against various mucin epitopes [143,144]. Currently, these aberrant glycoepitopes are being examined as potential targets for immunotherapies including the design of chimeric antigen receptor (CAR) T-cells against Tn on MUC1 [145,146]. These glycoepitopes are also commonly used as biomarkers for overall cancer progression, including CA19-9, CA15-3, DU-PAN-2, and CA-125 [147,148,149,150].

Aberrantly expressed oligosaccharides may also play a role in immune evasion. Expression of Tn and STn on MUC1 increase binding to the C-type macrophage galactose lectin (MGL), which is found on antigen-presenting cells [151,152,153]. MGL has been shown to dampen the adaptive immune response through reducing CD45-expressing T-cell proliferation and increasing T-cell death [154]. Expression of MUC16 is associated with immune protection by interfering with the formation of synapses between tumor cells and natural killer (NK) cells [155]. Expression of both MUC16 and MUC1 has also been shown to dampen the Toll-like receptor mediated immune response at ocular surfaces, and may play a role in tumor progression [156]. These effects may be dependent on a balance between the formation of truncated *O*-glycan structures and elongation, as Cosmc knockout cells exhibit increased sensitivity to both NK cells and cytotoxic T lymphocyte-mediated cell death [157].

Expression of particular glycan structures also plays critical roles in the metastatic spread of tumor cells through the body. Expression of the carbohydrate structures sialyl-Lewis^X^ and sialyl-Lewis^A^ on MUC1 enable the binding of MUC1 to both E-selectin and intercellular adhesion molecule (ICAM)-1 [158]. Likewise, glycosylated forms of MUC16 have been shown to bind both E- and L-selectin [159]. As interactions with selectins on endothelial cells and other cell types are critical for extravasation of immune cells from vasculature and subsequent trafficking through tissues, these interactions are proposed to similarly affect extravasation, invasion, and metastasis of tumor cells [160].

## 5. Summary

**Figure 5:**
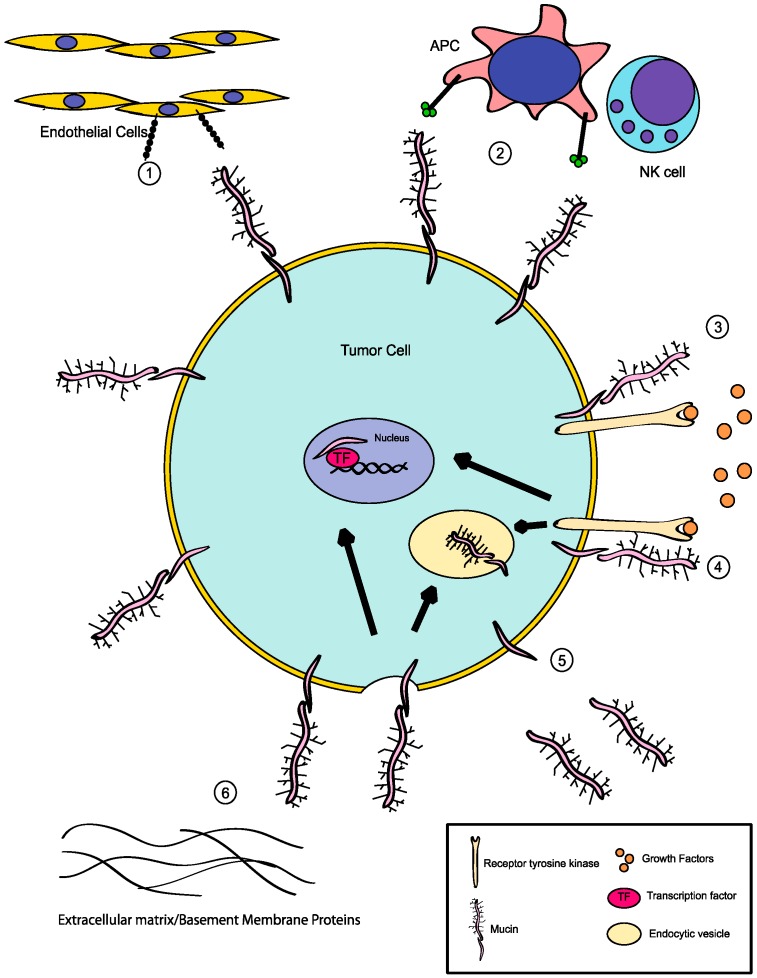
Impact of *O*-glycosylation on tumor cells. Aberrant *O*-glycosylation can induce a wide range of effects, including (1) alterations to interactions with microenvironment, such as increased association with endothelial cells and invasive behavior; (2) immune modulation through interaction with receptors expressed on antigen presenting cells, or other immune effector cells; (3) alterations to signaling complexes through the unmasking of domains critical for interaction with receptor tyrosine kinases or other effectors. This can result in either signaling at the surface or (4) alterations to cellular localization through endocytosis or translocation to the nucleus; (5) increased shedding of extracellular domains through exposure of cleavage sites. These events may also promote morphogenetic signaling; (6) disruption of interactions with extracellular matrix and basement membrane proteins resulting in migratory and invasive behaviors. APC: antigen-presenting cell; NK: natural killer.

*O*-linked glycosylation, one of the most abundant post-translational modifications observed within the cell, plays crucial roles in creating and modifying the structure and function of the molecules. Mucins and proteins with mucin-type domains are decorated with a wide variety of carbohydrate moieties, and it is imperative to better understand the functional outcome of alterations in glycosylation for the future study of cancer and other disease processes (Figure 5). Many studies have demonstrated that expression of truncated glycan structures modulates proliferative, migratory and invasive behaviors of tumor cells, in part by altering the interactions between the cell and the surrounding environment and by affecting important signaling pathways in cells. Future studies focused on glycosylation induced effects on signaling are needed to provide further insight into the manner by which these important post-translational modifications mediate crosstalk between cells and the surrounding microenvironment.

## Figures and Tables

**Table 1:** Deregulation of mucin expression in cancer.

Mucin	Cancer	Reference
MUC1, MUC4, MUC5AC, MUC6, MUC16	Pancreatic ductal adenocarcinoma	Remmers et al. [33]
		Hinoda et al. [34]
		Huang et al. [35]
		Haridas et al. [32]
		Higashi et al. [31]
MUC1, MUC2, MUC3, MUC4, MUC5AC, MUC5B, MUC6	Breast cancer	Ghosh et al. [36]
		Rakha et al. [37]
		Mukhopadhyay et al. [38]
MUC1, MUC2, MUC4, MUC5AC, MUC5B, MUC6, MUC17	Colon cancer	Terada et al. [39]
		Krishn et al. [24]
MUC1, MUC2, MUC4, MUC5AC, MUC6	Lung cancer	Awaya et al. [26]
		Kwon et al. [40]
MUC1, MUC4, MUC16	Ovarian cancer	Yin et al. [41]
		Chauhan et al. [42]
MUC1, MUC2, MUC4	Prostate cancer	Singh et al. [27]
		Osunkoya et al. [43]

**Table 2:** Confirmed phosphorylation sites in the cytoplasmic tails of MUC1, MUC4, and MUC16.

Mucin	Phosphorylation Site	Function (if Known)	Reference
MUC1	RRKNYGQLDI	N/A	Rikova et al. [80]
MUC1	PARDTYHPM	N/A	Gu et al. [81]
MUC1	PARDTYHPM	Interaction with AP1G, AP2M1, PIK3R1, p53 and increased cell motility	Singh et al. [77]
			Kinlough et al. [91,92]
			Kato et al. [93]
MUC1	YHPMSEYPT	N/A	Mertins et al. [79]
MUC1	PMSEYPTYH	N/A	Gu et al. [81]
MUC1	PMSEYPTYH	N/A	Mertins et al. [79]
MUC1	PMSEYPTYH	N/A	Rikova et al. [80]
			Gu et al. [81]
MUC1	HTHGRYVPP	N/A	Gu et al. [81]
MUC1	HTHGRYVPP	N/A	Rikova et al. [80]
			Gu et al. [81]
MUC1	VPPSSTDRS	Growth altered	Ren et al. [94]
MUC1	VPPSSTDRS	Inhibits interaction with β-catenin	Li et al. [95]
MUC1	SPYEKVSAG	Promotes association with β-catenin and inhibits association with GSK3β	Singh et al. [77]
			Li et al. [95]
MUC1	SPYEKVSAG	N/A	Mertins et al. [79]
MUC1	SSLSYTNP	Interaction with GRB2	Kinlough et al. [91]
MUC4 contains no confirmed sites of phosphorylation in the cytoplasmic tail			
MUC16	CPGYYQHLD	May regulate turnover	Das et al. [96]
			Akita et al. [97]

Phosphorylation site is highlighted in red for each mucin. The function (if known) is included for reference. AP1G: adaptor protein complex 1 gamma subunit; AP2M1: adaptor-related protein 2 mu 1 subunit; PIK3R1: phosphoinositide-3-kinase regulatory subunit 1; GSK3β: glycogen synthase kinase 3β; GRB2: growth factor receptor-bound protein 2.
